# Commentary: Without Values, Complexity is Reduced to Mathematics

**DOI:** 10.1111/jep.14263

**Published:** 2024-12-12

**Authors:** Trisha Greenhalgh

**Affiliations:** ^1^ Nuffield Department of Primary Care Health Sciences University of Oxford Oxford Oxfordshire UK

**Keywords:** causation, complexity science, health policy, medical ethics

## Abstract

This commentary on Sturmberg and Mercuri's paper ‘Every Problem is Embedded in a Greater Whole’ [1] argues that those authors have approached complexity from a largely mathematical perspective, drawing on the work of Sumpter. Whilst such an approach allows us to challenge the simple linear causality assumed in randomised controlled trials, it is itself limited. Mathematical complexity can explain nonlinearity and network effects but it cannot explain human values. It overlooks, for example, how science itself is historically and culturally shaped and how values‐driven misunderstandings and conflicts are inevitable when people with different world views come together to try to solve a problem. This paper argues that the mathematical version of complexity thinking is necessary but not sufficient in medical research, and that we need to enhance such thinking further with attention to human values.

## 1 Introduction: The Limits of Scientific Reductionism

Scholars from many different branches of philosophy have long decried the tendency for scientists and model‐makers to oversimplify reality. What does this latest contribution from Sturmberg and Mercuri [1] add? What does it fail to address?

The paper's introduction traces the origins of a deconstructionist, reduce‐and‐resolve approach to science back to the French philosopher Rene Descartes (1596–1650), who, for those at the back, famously separated the body from the mind and promoted the systematic, empirical testing of hypotheses to reduce doubt (a groundbreaking idea at the time). A particular focus of Descartes’ work, and that of many scientists in the Enlightenment (broadly, mid 17th to mid‐18th centuries), was the demonstration of causality. At the time, there were countless causal relationships still to be revealed. William Harvey showed, for example, in 1628, that the heart's pumping caused blood to circulate. Isaac Newton, in 1687, published his account of causal relationships between force, mass, acceleration and gravity, leading to his laws of motion that were foundational to modern physics. Benjamin Franklin, in 1750, experimented with kites and thunder clouds (Google it—this is hypothesis‐testing at its best) to demonstrate a causal relationship between electric charge and lightening, leading to the invention of the lightning rod.

It is no accident that these well‐publicised examples of scientific breakthroughs all involved a mechanical or electrical relationship between objects or particles. If A does X, then Y happens to B. Simples. Direct, linear causality can sometimes be shown in complex fields like public health too. As Sturmberg and Mercuri remind us, Edward Jenner's discovery of the preventive effect of cow pox on the development of smallpox (1796), Semmelweiss' revelation that handwashing prevented puerperal sepsis (1847) and John Snow's demonstration of the waterborne nature of cholera (1854) were *causal* discoveries which transformed practice in their own right and set the stage for subsequent addition of more pieces of the causal jigsaw—namely, the identification of specific organisms and the development of antimicrobials. If you pick your examples carefully, you can make the case that medical science was built, brick by brick, by solving deconstructed problems and finding out, in each case, what happens to B if A does X.

But even in the 17th and 18th centuries, there were counterexamples of phenomena that could *not* be explained in terms of simple causality. As Sturnberg and Mercuri point out, leading the push‐back against what is sometimes called (after Descartes) ‘Cartesian’ reductionism were Johann Wolfgang von Goethe and Alexander von Humboldt. These German polymaths both observed, at the turn of the 19th century, that the whole cannot be fully known solely by knowing its constituent parts when it involves living things in their natural environment (Goethe, e.g., noticed that plants growing at higher altitude were smaller than those at lower altitude, leading him to hypothesise about differences in key environmental nutrients). It is worth noting that, as the 19th century German philosopher Wilhelm Dilthey might have added, the same goes for human beings in their social worlds. Dilthey developed the important concept of ‘Verstehen’ (‘world‐view’), analysing historical texts to understand the world‐view of past societies. He showed how people's beliefs, values, and customs shaped their understanding of the world and their place within it.

## 2 Thinking About Complexity

### 2.1 Sumpter's Taxonomy: A Mathematical Take on Complexity

To explore how we might study the individual in their wider environment, Sturmberg and Mercuri summarise Sumpter's ‘Four ways of thinking’ [2]:

- [Basic] *statistical thinking*, according to this taxonomy, is about correlation and probabilistic prediction. It's often helpful, but it can mislead because not everything can be reduced to numbers and because unmeasured variables may produce hidden biases.
- *Interactive thinking* considers how a relationship between two or more variables changes over time. It illustrates dynamic change—for example, how population numbers wax and wane—but is not designed to incorporate all the variables that might influence those patterns.
- *Chaos thinking* refers to a mathematical phenomenon in which the outputs of a system (such as a neural network) are heavily dependent on initial conditions. A small input sometimes produces a large impact and vice versa.
- *Complexity thinking* considers the system as a whole and the dynamics of its evolution. The system evolves as relationships between nodes in the network shift. The individual is nested in a wider context and influenced by both historical and present‐day connections. As with chaos thinking, a standard input will not generate a specific output. You cannot step into the same river twice.

While Sumpter's taxonomy is illuminating, it is a mathematician's view of complexity which under‐emphasises how and why *humans* are complex. The ‘chaos’ in Sumpter's chaos thinking, for example, is a mathematical chaos, and whilst it can serve as a *metaphor* for social chaos, its explanatory power for the latter is limited. Similarly, while complexity thinking as expounded by Sumpter will explain very well how a termite mound, the immune system or the distribution of plants on an Alpine hillside change over time, it is less suited to explaining the complexities of humans in their evolving societies.

### 2.2 Human Complexity: The Importance of Values

Human societies are complex not merely because societies contain a lot of people and a lot of connections but also because people have ambitions, values, moral causes about which they may feel passionately, and things at stake. To a greater or lesser extent, they care about human rights, equity and fairness. They do things that are culturally meaningful, and they tend not to do things that lack meaning for them. They have personal and professional standards which give impetus to their work and produce red lines that they will not cross. As Dilthey showed back in the 1800s, these human values and perspectives are historically and culturally generated and as hard to change as the thinness of the air at altitude.

Why does this human element require us to update our taxonomy of complexity? Because—among other reasons—deeply‐held human values and standards generate mistrust, misunderstanding and conflict [3]. Values are often the reason, for example, why clinicians ‘resist’ the introduction of new technologies or ways of working (because, rightly or wrongly, they perceive that the standards of excellence they were taught cannot be upheld in the proposed new system) [4, 5]. Values explain why, in the USA, gender‐affirming care is recommended as an evidence‐based therapy [6] but also criminalised in some states [7]. Values explain why medical‐scientific debate during the COVID‐19 pandemic became polarised along libertarian lines, with lockdowns, masks and vaccines all depicted by some as evidence‐based public health measures [8, 9, 10] and by others as of limited value and an unjustified infringement of individual freedom [11, 12, 13]. A purely mathematical take on complexity will fail to acknowledge or address these and other values‐based conflicts which characterise *human* complex systems.

This cuts to the heart of my concern about Sturmberg and Mercuri's presentation of how different kinds of research contribute differently to the reduction of uncertainty. Table 1 in their paper is a helpful summary of study designs in epidemiology but does little more than add a row depicting mathematical relationships (one‐to‐one, one‐to‐many, many‐to‐one and many‐to‐many) to repackaged conventional thinking. The pragmatic randomised controlled trial, for example, designed to produce a more clinically useful balance between internal and external validity, has been around for decades and its epistemic contribution is well‐documented [14]. The continuum from ‘complete ignorance’ to ‘absolute certainty’ (with a gap to acknowledge that some things are unknowable) is, I would argue, epistemologically naïve as it implies a single, external truth towards which scientific inquiry inexorably progresses. This linear visualisation seems to reinforce rather than challenge reductionist scientific thinking, though I acknowledge that all visualisations are oversimplifications.

## 3 The Individual and the Whole: How to Research Context

### 3.1 Accounting for Multiple Variables

The clinical examples in the second half of their paper contain (perhaps of necessity) much epidemiological (and, in some cases, biochemical) detail. The message, which is hard to divine amongst all this detail, is that there is a granularity to all diseases in the form of multiple relevant variables, and that crude study designs (in which, e.g., a sample is stratified only by age, gender and disease severity) miss this granularity because most variables go unmeasured or inadequately analysed.

As Figure 4 in Sturmberg and Mercuri's paper shows, people with cardiovascular disease can be grouped into clusters using deep learning techniques. People in some of these clusters are less likely to have had previous heart events; some are more likely to take their medication; some are more likely to have certain genetic markers—and so on. Likewise for type 2 diabetes. In glioblastoma multiforme, the granularity is in the immunological markers, which (using deep learning) divide broadly into four overlapping clusters. The future seems bright for ‘targeted prevention’ and ‘targeted pharmacotherapeutics’, in which therapy decisions take account of disease granularity [1]. This argument makes perfect sense, but followed to its logical conclusion, the best we can hope for is doing more of ‘the thing right’—more randomised controlled trials (pragmatic of course), but with samples stratified by multiple pre‐identified variables. And, perhaps, more basic science research to identify the key causal mechanisms that can inform the design of therapeutic trials [15].

The section of Sturmberg and Mercuri's paper, on ‘Context beyond the biomedical’, presents data (see in particular Table 2) to support the argument that environmental variables (such as socioeconomic status) are at least as significant as biomarkers (such as serum cholesterol) in the development of diseases (such as coronary artery disease). I'm not sure Sir Michael Marmot, who published the Whitehall II study in 1991, would be surprised at this [16], though the deep learning methodology which affirms those early findings is relatively new. I also accept the point that research in coronary artery disease continues to be focused on changing biomarkers and individual behaviours rather than on finding how to influence the structural conditions in our unequal society which predispose some people to smoking, obesity and high levels of psychological stress.

I also accept the argument (which, by and large, is not new) that shifting from a purely statistical way of thinking to one that embraces the principles of complexity (consider context; look for interactions and networked relationships in the system as a whole; accept that not everything is predictable, and so on) will serve us well when seeking solutions for complex diseases that have complex chains of causation.

### 3.2 Beyond Variables: Factoring in Values to the Clinical Trial Paradigm

I do not, however, accept that the techniques listed in the previous paragraph and others listed under ‘Finding the right approach’ [1] are all that's needed, because all these stem from a concept of complexity that is essentially mathematical. If, instead of mathematical complexity, we add *human* (including cultural and historical) complexity to the mix here, some different insights emerge.

Most importantly, science progresses not brick by culturally neutral brick but in *paradigms* [17]. A paradigm is a world view shared by a group of scientists; it generates an unfolding (and incrementally changing) research tradition based on shared assumptions and values about which questions are important and which methods are deemed high‐quality. The many‐to‐many mathematical status of the pragmatic randomised controlled trial depicted by Sturnberg and Mercuri is, perhaps, of less significance than the gradual evolution of the clinical trial paradigm to reduce hidden biases [18] such as the under‐representation of women, racial and ethnic minorities, older people, pregnant people, people whose condition is complicated by multimorbidity, people with rare diseases, and people with mental health conditions or learning difficulties [19, 20, 21, 22, 23, 24].

Similarly, the development and use of patient‐reported outcome measures [25], though not without its critics [26, 27, 28, 29], has helped to ensure that trials better reflect what matters to patients. These changes were driven as much by human values as by concerns about statistical representativeness: it is a *human right* to have one's treatment determined by findings from well‐conducted clinical trials and for ‘success’ to be measured in a currency that has meaning.

### 3.3 New Paradigms: Challenging Western Ways of Knowing

More radically, the epidemiological/clinical trials paradigm depicted in Sturnberg and Mercuri's Table 1 has been challenged as an example of Western ways of knowing [30, 31, 32, 33]. According to this argument, evidence‐based medicine's hierarchy of evidence, far from being a self‐evident truth, is the product of historical and cultural influences which prioritise particular assumptions and approaches and which both produce and reproduce racial, cultural and economic inequities. This occurs, for example, through the dominance of the field by researchers from the Global North, the alignment of academic research with the commercial interests of pharmaceutical companies, the use of extractive methods of data gathering which pay scant attention to the needs or values of the communities being studied, the devaluing of the subjective and the collective, the prohibitive costs of publishing in high‐impact journals, and the tendency to emphasise individual behaviour change rather than acknowledge and address society's structural inequities.

From this more radical perspective, including more under‐researched groups in clinical trials is equivalent to shifting the deckchairs on the Titanic since it will not alter the fundamental historical power imbalances in what counts as knowledge. A full analysis of decolonialist critiques of epidemiology and clinical trials, and wider work on epistemic justice (in which the knowledge generated by less powerful groups is rejected as illegitimate by more powerful groups) [34], is beyond the scope of this commentary. My point here is that a human (as opposed to mathematical) approach to complexity would place such critiques front and centre.

Is it fair to criticise Sturmberg and Mercuri for not extending their analysis to embrace how cultural and historical forces shape and constrain the thinking and our collective practices of clinical researchers? Perhaps not, on the grounds that they are making a legitimate point about how mathematical complexity changes the rules for epidemiological studies. But perhaps it is, since their antireductionist call for more complexity thinking is justified specifically with reference to the distinction between ‘doing the thing right’ and ‘doing the right thing’ popularised by management guru Peter Drucker [35]. It seems to me that the argument presented is focused, conservatively, on the principles of study design (i.e., *how* a thing—in this case epidemiology—should be done) rather than on any over‐arching *moral* questions about *what* should be done. By excluding value complexity [3] from their theoretical frame, and by largely ignoring prevailing debates about (e.g.,) inclusivity and decolonialism, it misses an important opportunity to rise above questions of method and ask what *is* the right thing to do.

## 4 Conclusion

I came to this paper as someone who has long been sympathetic to complexity thinking and has called out conventional epidemiologists for talking the language of complexity but failing to embrace its fundamental principles [36]. In this new paper, Sturmberg and Mercuri show that, unlike many biomedical researchers, they understand the principles of complexity thinking. But their analysis is limited to drawing (as many medical scholars do) on an overly mathematical version of such thinking.

In this commentary, I hope that I have highlighted the many strengths of their paper while also inviting those authors and their readers to extend their concept of complexity to include *human* complexity—that is, the complexity which arises when values clash, meanings are contested, and people get into conflict about what matters to them and what is at stake for them.

The question of what would count as the ‘right approach’ to research if we expanded our definition of complexity thinking to include human (i.e., value‐driven) complexity requires a paper of its own (or even an entire edition of this journal), which the editors of this journal might wish to commission.

Briefly, such an approach would place greater emphasis on human relationships (including the relationships between research teams and the communities they seek to study), include an essential phase of facilitated dialogue with those communities before any research could begin, require respect for non‐epidemiological ways of knowing (hence, at the very least, would upgrade the value of qualitative and co‐produced research and instil epistemological humility in researchers), and accept that conflict, misunderstanding and mistrust are always ‘in the mix’ when groups of people with different world views come together to address a problem.

In sum, the perspective on complexity expounded by Sturmberg and Mercuri is welcome but not sufficient. Let's take the debate to the next level.

## Conflicts of Interest

TG is a member of Independent SAGE. She holds a grant from UKRI to study the philosophical aspects of evidence.

## Data Availability

This commentary draws entirely on published papers in the public domain.
